# Association between the single nucleotide variants of the mitochondrial cytochrome B gene (*MT-CYB*) and the male infertility

**DOI:** 10.1007/s11033-022-07200-y

**Published:** 2022-02-03

**Authors:** Mayyas Saleh Jaweesh, Mohamad Eid Hammadeh, Fatina W. Dahadhah, Mazhar Salim Al Zoubi, Houda Amor

**Affiliations:** 1 Department of Obstetrics & Gynaecology, Saarland University, Homburg, Saar Germany; 2 Department of Basic Medical Sciences, Faculty of Medicine, Yarmouk University, Irbid, 21163 Jordan

**Keywords:** Idiopathic infertility, mtDNA mutation, *MT-CYB*, Polymorphisms

## Abstract

**Background:**

Idiopathic male infertility can be attributed to genetic predispositions that affect sperm performance and function. Genetic alterations in the mitochondrial DNA (mtDNA) have been linked to certain types of male infertility and abnormal sperm function. Mutations in the mitochondrial cytochrome B (*MT-CYB*) gene might lead to some deficiencies in mitochondrial function. Thus, in the current study, we aimed to investigate the effect of mutations in the *MT-CYB* gene on sperm motility and male infertility.

**Methods and results:**

Semen specimens were collected from 111 men where 67 men were subfertile and 44 were fertile. QIAamp DNA Mini Kit and REPLI-g Mitochondrial DNA Kit from QIAGEN were used to isolate and amplify the mitochondrial DNA. Followed by PCR and Sanger sequencing for the target sequence in the *MT-CYP* gene. Sequencing of the *MT-CYB* gene revealed a total of thirteen single nucleotide polymorphisms (SNPs). Eight SNPs were non-synonymous variant (missense variant) including: rs2853508, rs28357685, rs41518645, rs2853507, rs28357376, rs35070048, rs2853506, and rs28660155. While five SNPs were Synonymous variant: rs527236194, rs28357373, rs28357369, rs41504845, and rs2854124. Among these SNPs, three variants showed a significant difference in the frequency of the genotypes between subfertile and fertile groups: rs527236194 (T15784C) (*P* = 0.0005), rs28357373 (T15629C) (*P* = 0.0439), and rs41504845 (C15833T) (*P* = 0.0038). Moreover, two SNPs showed a significant association between allelic frequencies of rs527236194 (T15784C) (*P* = 0.0014) and rs41504845 (C15833T) (*P* = 0.0147) and male subfertility.

**Conclusion:**

The current study showed a significant association between the *MT-CYB* gene polymorphisms and the development of male infertility. In particular, rs527236194, rs28357373 and rs41504845 variants were found to be the most related to the subfertility group. Further studies on larger and other populations are required to reveal the exact role of this gene in the development of male infertility. In addition, functional studies will be helpful to elucidate the molecular impact of the M*T-CYP* polymorphisms on mitochondrial function.

## Introduction

Infertility is one of the common health problems with social and economic impacts on couples’ lives. Studies regarding the diagnosis and treatment of infertility have increased dramatically since the first in-vitro fertilization (IVF) baby was born in July 1978. All infertility studies are aiming to increase the efficiency of the diagnosis and most importantly increasing the success rate of fertility [1]. Infertility affects around 15% of couples globally [2], and genetic abnormalities are believed to account for approximately 15–30% of the malefactor of infertility [3]. Pieces of evidence are suggesting that male infertility has a genetic link by influencing different physiological processes including hormonal homeostasis, spermatogenesis, and sperm quality [3]. Thus, an understanding of the genetic basis of reproductive failure is important to manage infertile couples.

Mitochondrial mutations are very common etiological pathogenic factors in energy-demanding organs such as muscles and the nervous system and are related to musculoskeletal and neurodegenerative disorders [4]. In addition, genetic alteration in the mtDNA may have significant effects on normal spermatogenesis and subsequently on fertilization [5, 6]. Several reports have shown that mitochondrial dysfunction could lead to complete or partial spermatogenesis arrest and be an important causative factor in male infertility [7]. Therefore, studying and analyzing the alterations in the sperm mitochondrial DNA genes, which encodes for the oxidative phosphorylation complexes and are responsible for the production of energy (ATP) required for normal sperm motility [3], could help to understand the unknown causes of male infertility.

Moreover, single nucleotide polymorphisms (SNPs) in the mitochondrial genome were linked to several disorders in humans [8]. The mitochondrial DNA encodes for several polypeptides of the respiratory chain which are located in the inner membrane of the mitochondria and responsible for the oxidative phosphorylation cycle. There is accumulating data showing that SNPs in the mtDNA might have a main role in determining male fertility [9]. For instance, impaired sperm motility (Asthenozoospermia) is usually a foremost cause of male infertility.

Asthenozoospermia is caused by a variety of factors, including ultrastructural abnormalities of sperm, abnormal liquefaction of semen, functional deficiencies, antisperm antibodies, varicocele, endocrine abnormalities, or other physical and chemical factors [8]. Also, sperm motility is a result of complex molecular events. This includes the oxidation of energy substrates, then the phosphorylation of the proteins involved in ionic signal transduction through the plasma membrane and finally the conversion of chemical energy into mechanical energy in the sperm axoneme. Therefore, the normal structure and function of the mitochondria are crucial for sperm motility and male fertility [10]. Nevertheless, during oocyte fertilization, sperms require a large amount of energy for the movement of their flagella. Therefore, about 100 mitochondria are present in the midpiece of each mature human spermatozoon to provide energy quickly and effectively for sperm motility [11]. Thus, the integrity of the mitochondrial DNA is important for the normal conduction of the electron transport chain and OXPHOS complexes to produce enough amount of adenosine triphosphate (ATP) to fuel sperm motility. Abnormal sperm motility could be linked to multiple deletions in the mitochondrial DNA [12]. These deletions are related to the loss of the vital proteins that are forming the electron transport chain and OXPHOS complexes which eventually lead to a shortage in ATP production. ATP molecules are fuel sperm to reach the fallopian tubes where the fertilization takes place [3]. Some studies have illustrated a positive correlation between respiratory chain enzymatic activities and sperm motility, indicating that functional changes in mitochondrial might cause asthenozoospermia [13].

The mitochondrially encoded Cytochrome B (*MT-CYB*) gene is located at position 12 of the mitochondrial genome and encodes for cytochrome B protein, one of the respiratory chain proteins Ubiquinol Cytochrome c Reductase (UQCR, Complex III or Cytochrome bc1 complex) [14]. Mutations in *MT-CYB* have been related to several disorders, resulting in a deficiency in the process of catalyzing electron transfer through the mitochondrial respiratory chain [14]. Thus, in the current study, we purpose to find out the possible effect of genetic alterations in the *MT-CYB* gene on sperm motility and male infertility.

## Materials and methods

### Patient’s recruitment and sample collection

The study population included 111 individuals. Semen samples were collected from individuals by masturbation using a sterile container after 3–5 days of abstinence. Demographic and clinic pathological data were collected such as; age, health status, history of varicoceles and presence of chronic diseases. The fertile males included in this study were healthy men of reproductive age (25–55 years old), free from chronic diseases such as diabetes and blood pressure, did not have varicoceles, not exposed to chemotherapy or radiotherapy, did not do any surgical procedure in the reproductive tract, with no hypogonadotropic hypogonadism (hormonal disorder) and no genetic disorders (Klinefelter's syndrome or Y-chromosome microdeletion). The control group included 44 fertile men with a normal semen analysis (Concentration: 15 × 10^6^ spermatozoa/ml, total motility: ≥ 40%, progressive motility: ≥ 32%, and normal morphology: ≥ 4%), while the subfertile group included 67 non-fertile men with abnormal motility (< 40%) and abnormal progressive motility (< 32%).

The name of the committee that ethically approved this study is: Jordanian Royal Medical Services-Human Research Ethics Committee. The approval number is: F3/1/Ethics Committee/9126.

### Semen analysis

After the collection, samples were allowed to liquefy at room temperature (RT) for 30 min then semen analysis was done according to WHO 2010 guidelines including sperm concentration, motility, and morphology. Then, semen samples were prepared using Pureception density gradient media (45%/90%), centrifuged 1000×*g* for 20 min, then the pellet was washed using Global Total HEPES media with HSA (Cooper surgical, Denmark) and centrifuged 1200×*g* for 10 min then the final pellets were resuspended in 0.5 ml of culture media and kept frozen in − 80 °C to be later used for DNA extraction.

### Mitochondrial DNA extraction

The isolation of the mitochondrial DNA from sperm was performed in two steps. Firstly, the QIAamp DNA Mini Kit (QIAGEN, Germany) was used to isolate the mitochondrial DNA (The extraction was done according to the instructions recommended by the kit). For the DNA extraction procedure, the sperm pellet was digested by adding 20 μL of protease K to 200 μL of washed semen samples, mixed then incubated overnight at 56 °C. The next day, add 200 μL of the AL Buffer to the mixture. Then, 200 μL absolute ethanol was added and the mixture was mixed and centrifuged at 6000×*g* for 1 min. Next, 500 μL washing buffer AW1 was added to the pellet and centrifuge at 6000×*g* for 1 min. After that, 500 μL of washing buffer AW2 was added, and the mixture was centrifuged at full speed (20,000×*g* for 3 min. Finally, 200 μL of the last buffer was added and then centrifuged at 6000×*g* for 1 min.

Secondly, the REPLI-g Mitochondrial DNA Kit (QIAGEN, Germany) was used to isolate and amplify only mitochondrial DNA from the samples (The extraction was done according to the instructions recommended by the kit).

The MT-DNA was amplified by adding 10 μL of RNase-Free water to the 10 μL of the extracted DNA sample into a microcentrifuge tube to adjust the sample volume to 20 μL. Then, 29 μL of the amplification mixture was added to the tubes containing the DNA samples, incubated for 5 min using the thermocycler at 75 °C and allowed to cool down to room temperature (15–25 °C).

The REPLI-g Midi DNA polymerase was thawed on ice and 1 μL of it was added to the DNA mixture from the last step and incubated at 33 °C for 8 h. Finally, the enzyme was inactivated by heating the sample for 3 min at 65 °C.

The quantity and quality of the isolated DNA samples were measured and checked using the Nanodrop spectrophotometer ND-2000c (Thermo Scientific, USA). An optical density ratio of 260/280 of 1.8 or more only was chosen. After that, the amplified DNA was Kept at − 80 °C for later use.

### Polymerase chain reaction (PCR)

Primers used for the amplification were designed using the UCSC website and the PRIME 3 software and were ordered from the Microsynth Seqlab company (Göttingen, Germany) (Table 1).

**Table 1:** Forward and reverse primers for *MT-CYB* gene

Gene	Primer direction	Sequence (5′→3′)	Product length
MT-CYB	MT-CYB-F	CGGACTACAACCACGACCAA	1246 bp
	MT-CYB-R	TCCGGTTTACAAGACTGGTGT	

A 30 µL PCR reaction mixture was prepared using MyTaq^TM^HS Red Mix Kit (Bioline, UK) according to the manufacturer’s instructions. Then, the amplification was performed on the thermocycler (C1000™ Thermal cycler, Bio-Rad, United States) under the following conditions: The initial denaturation was carried out at 95 °C for 3 min, followed by 35 cycles of denaturation (95 °C × 40 s), annealing (57 °C × 30 s), and extension (72 °C × 1 min) took place. Finally, the final extension was done at 72 °C for 5 min. Five μL of the PCR products for each sample was run on gel electrophoresis using 1.5% agarose along with DNA Ladder (0.1–10.0 kb) (NE Biolabs, USA). It was run at 75 V for 1 h in 1× TAE buffer then visualized by Molecular Imager® Gel Doc™ XR (BIO-RAD, USA).

### Sanger sequencing

Samples were purified and sequenced using the Sanger Sequencing method by Seqlab (Sequencing Laboratories, Göttingen, GmbH). Mutation surveyor software was used to identify the SNPs of the *MT-CYB*. Thereafter, all resulting SNPs were genotyped using finch TV.

### Statistical analysis

Statistical analyses were performed to analyze genotypes and allele frequencies between case and control groups using the chi-square test and Fischer’s exact test using the SPSS program version 23. Odds ratios were calculated together with their 95% confidence intervals (CI). For mean comparison, the t-test was used for the calculation. A *P*-value of < 0.05 was statistically significant.

## Results

The study population showed no statistically significant difference in age between the fertile and subfertile groups *(P* = 0.225). In addition, the studied semen parameters were significantly higher in the fertile group compared to the subfertile group: sperm concentration (78.5 × 10^6^/ml vs 28 × 10^6^/ml), total motility (67.5% vs 20%), and normal morphology (24.5% vs 15%), *P* < 0.0001 (Table 2). The direct sequencing of the cytochrome B mitochondrial DNA gene (*MT-CYB*) identified a total of 13 single nucleotide polymorphisms (SNPs) in the study population. All the detected SNPs have been reported in the national center for biotechnology information database (NCBI).

**Table 2:** Comparison of the semen analysis parameters between the control group (Fertile) and case groups (Sub-fertile)

Parameter	Fertile (n = 44) Median	Subfertile (n = 67) Median	Mini–max	*P*-value (*t-test*)
Age (years)	34	34	25–55	0.225
Sperm concentration (10^6^/ml)	78.5	28	0.4–185	< 0.0001
Total motility (%)	67.5	20	2–90	< 0.0001
Normal morphology (%)	24.5	15	0–30	< 0.0001

Among the detected single nucleotide polymorphisms (SNPs), three of polymorphic variants showed a significant difference in the genotype’s frequency test between subfertile and fertile groups: rs527236194 (*P* = 0.0005), rs28357373 (*P* = 0.0439), and rs41504845 (*P* = 0.0038) (Table 3). For the allele’s frequency test, two SNPs were significant: rs527236194 (*P* = 0.0014) and rs41504845 (*P* = 0.0147) (Table 4). Moreover, eight SNPs were non-synonymous variants (missense variant) including: rs2853508, rs28357685, rs41518645, rs2853507, rs28357376, rs35070048, rs2853506, and rs28660155 and five SNPs were synonymous variants: rs527236194, rs28357373, rs28357369, rs41504845, and rs2854124 (Table 3).

**Table 3:** Genotypes of MT*-CYB* polymorphisms between subfertile patients and fertile individuals

SNP	Contig position	Protein position	Amino Acid change	Genotype	Subfertile (N)	Fertile (N)	*P*-value
RS2853508 A>G	15326	Thr194Ala	Missense variant	AA	0	0	N/A
				AG	0	0	
				GG	67	44	
RS28357685 G>A	15110	Ala122Thr	Missense variant	GG	0	0	1.000
				GA	2	1	
				AA	65	43	
RS527236194 T>C	15784	Pro346	Synonymous variant	TT	67	40	**0.0005**
				TC	0	0	
				CC	0	4	
RS41518645 G>A	15257	Asp171Asn	Missense variant	GG	62	41	1.000
				GA	0	0	
				AA	5	3	
RS2853507 G>A	15317	Ala191Thr	Missense variant	GG	67	43	0.1560
				GA	0	0	
				AA	0	1	
RS28357373 T>C	15629	Leu295	Synonymous variant	TT	63	44	**0.0439**
				TC	1	0	
				CC	3	0	
RS28357376 A>G	15824	Thr360Ala	Missence variant	AA	66	44	0.5193
				AG	0	0	
				GG	1	0	
RS35070048 A>G	15311	Ile189Val	Missence variant	AA	66	44	0.5193
				AG	0	0	
				GG	1	0	
RS28357369 A>G	15244	Gly166	Synonymous variant	AA	64	43	0.7060
				AG	1	0	
				GG	2	1	
RS41504845 C>T	15833	Leu363	Synonymous variant	CC	61	44	**0.0038**
				CT	1	0	
				TT	5	0	
RS2853506 A>G	15218	Thr158Ala	Missence variant	AA	67	43	0.1604
				AG	0	0	
				GG	0	1	
RS2854124 C>T	15136	Gly130	Synonymous variant	CC	67	43	0.3964
				CT	0	1	
				TT	0	0	
RS28660155 T>C	14871	Ile42Thr	Missence variant	TT	63	43	0.0909
				TC	0	1	
				CC	4	0

**Table 4:** Alleles frequency of MT-CYB polymorphisms between subfertile patients and fertile individuals

SNP	Contig position	Protein position	Alleles	Subfertile (N,%)	Fertile (N,%)	OR (95% CI)*	*P*-value
RS2853508 A>G	15326	Thr194Ala	A	0 (0%)	0 (0%)	N/A	N/A
			G	134 (60%)	88 (40%)		
RS28357685 G>A	15110	Ala122Thr	G	2 (1%)	1 (0%)	1.318 (0.1177 to 14.769)	0.8221
			A	132 (59%)	87 (39%)		
RS527236194 T>C	15784	Pro346	T	134 (60%)	80 (36%)	28.404 (1.617 to 499.07)	**0.0014**
			C	0 (0%)	8 (4%)		
RS41518645 G>A	15257	Asp171Asn	G	124 (56%)	82 (37%)	0.9073 (0.3175 to 2.593)	0.8559
			A	10 ( 4%)	6 (3%)		
RS2853507 G>A	15317	Ala191Thr	G	134 (60%)	86 (39%)	7.775 (0.3685 to 164.02)	0.3044
			A	0 (0%)	2 (1%)		
RS28357373 T>C	15629	Leu295	T	127 (57%)	88 (40%)	0.09605 (0.005412 to 1.705)	0.0741
			C	7 (3%)	0 (0%)		
RS28357376 A>G	15824	Thr360Ala	A	132 (59%)	88 (40%)	0.2994 (0.01419 to 6.316)	0.6707
			G	2 (1%)	0 (0%)		
RS35070048 A>G	15311	Ile189Val	A	132 (59%)	88 (40%)	0.2994 (0.01419 to 6.316)	0.6707
			G	2 (1%)	0 (0%)		
RS28357369 A>G	15244	Gly166	A	129 (58%)	86 (39%)	0.6000 (0.1138 to 3.164)	0.8292
			G	5 (2%)	2 (1%)		
RS41504845 C>T	15833	Leu363	C	123 (55%)	88 (40%)	0.06067 (0.003526 to 1.044)	**0.0147**
			T	11 (5%)	0 (0%)		
RS2853506 A>G	15218	Thr158Ala	A	134 (60%)	88 (40%)	7.599 (0.3603 to 160.28)	0.3130
			G	0 (0%)	2 (1%)		
RS2854124 C>T	15136	Gly130	C	134 (60%)	87 (39%)	4.611 (0.1856 to 114.58)	0.8319
			T	0 (0%)	1(0%)		
RS28660155 T>C	14871	Ile42Thr	T	126 (57%)	87 (39%)	0.1810 (0.02223 to 1.474)	0.1503
			C	8 (4%)	1 (0%)		

*N/A* non-applicable

## Discussion

The DNA of the mitochondria is semi-autonomous and does not undergo recombination. It undergoes a rapid replication process without efficient proofreading or DNA repair mechanisms. Because of its distinctive replication mechanism and its location in a highly oxidative environment, the mitochondrial DNA (mtDNA) mutates at a rate 10–20 times higher than that of nuclear DNA [3]. Expectedly, alterations in the mitochondrial DNA can cause diseases through various mechanisms such as interference with the oxidative phosphorylation (OXPHOS) function which is the most significant interruption [15]. Such mutations may cause serious disorders from minor manifestations of clinical presentations to life-threatening disorders of mitochondrial pathophysiology conditions [16].

MtDNA molecules of human sperm are prone to oxidative damage and mutations which have been shown to play an important role in male infertility [17]. It is well known that mtDNA is a major motor of ATP production in sperm through the OXPHOS process by encoding for protein subunits in the respiratory chain. OXPHOS process is also the position of reactive oxygen species (ROS) production and DNA ROS-induced damage [18]. During the process of spermatogenesis, a defect in mitochondrial DNA could increase the chance of free radical formation which creates an environment of oxidative stress that might disturb sperm development and function [19]. Also, sperm motility is highly dependent on ATP biosynthesis, which is carried out by the mitochondrial OXPHOS system, and consequently, sperm mtDNA variations result in functionless proteins [20].

In the current study, we identified 13 SNPs in *MT-CYB* gene in study population: five SNPs (rs527236194, rs28357373, rs28357369, rs41504845, and rs2854124) were synonymous variants and eight SNPs were non-synonymous variants (missense variant) including: rs2853508 (Thr194Ala), rs28357685 (Ala122Thr), rs41518645 (Asp171Asn), rs2853507 (Ala191Thr), rs28357376 (Thr360Ala), rs35070048 (Ile189Val), rs2853506 (Thr158Ala), and rs28660155 (Ile42Thr) and five SNPs were synonymous variant: rs527236194, rs28357373, rs28357369, rs41504845, and rs2854124.

The current findings showed an association between the occurrence of mutations in the *MT-CYB* gene and men’s infertility. In particular, rs527236194 (*P* = 0.0005), rs28357373 (*P* = 0.0439), and rs41504845 (*P* = 0.0038) demonstrated a statistically significant association with men infertility. Moreover, in the allelic frequency test, two synonymous SNPs (rs527236194 (*P* = 0.0014) and rs41504845 (*P* = 0.0147)) revealed a significant correlation with men’s infertility. The substitution mutations in the above-mentioned codons were synonymous. In particular, the rs527236194 variant alters [CC**T**] to [CC**C**] at position 15784 (Proline). While the rs28357373 variant changes [**T**TA] to [**C**TA] at position 15629 (Leucine) and rs41504845 variant changes [**C**TA] to [**T**TA] at 15833 (Leucine) (NCBI).

Despite the synonymous coding variant SNPs, such genetic alterations might be related to male infertility if we understand their role in gene regulation. Usually, synonymous changes do not affect the structure and function of the protein. However, previous research proposed that codon bias could be a mechanism for controlling the levels of gene expression [21]. As the choice of codon or which codon is preferred depends on which codon is translated more rapidly, promptly, and effortlessly, the high number of synonymous changes can decrease the overall translation rate. Thus, due to code degeneracy, many synonymous variations may not change amino acids. However, it may influence the efficiency of the translation machinery and thus may reduce the rate of production of ATP [22]. Therefore, functional studies are required to understand the exact role of these synonymous alterations on gene function and regulation.

At the molecular level, the respiratory complex III subunit is only encoded by the *MT-CYB* gene. Previous studies reported an association between the *MT-CYB* gene polymorphisms and certain disorders. For instance, rs2853508 was found to be associated with breast cancer [23]. Moreover, rs41518645 has been suggested to be one of the primary mutations causing Leber hereditary optic neuroretinopathy (LHON) [24]. In addition, the rs2853506 polymorphism (A15218G) has been associated with epileptogenesis [25].

The present results are in accordance and support the previous findings of the role of *MT-CYP* gene alteration in male infertility. For instance, A study reported that *MT-CYB* and *MT-ATP6* gene deletions and mutations in sperm could affect sperm motility [26]. In other studies, genetic alterations in the *MT-CYB* gene were proposed to be associated with the development of male infertility. For instance, three polymorphic sites in the *MT-CYB* gene (G15301A, A15326G and A15487T) are thought to be related to men’s fertility. The researchers also reported a novel synonymous substitution mutation (A to G) at position 15,472 in the *MT-CYB* gene in an infertile individual [27].

Point mutations, deletions and the presence of a particular haplogroup of mtDNA have been related to poor sperm quality [3]. For instance, mtDNA deletions have been associated with Asthenozoospermia and men’s infertility in different studies [3]. Many other studies in various populations have been conducted to elucidate the molecular bases and genetic factors that might be related to male infertility and sperm quality [28]. However, the etiology of idiopathic male infertility is not fully understood. Therefore, genetic analysis of the mtDNA can be a helpful tool to reveal the role of the mtDNA alteration in a subgroup of infertile men.

Nevertheless, the mitochondrial DNA mutations and their relationship to male infertility have been extensively studied, but still, there are discrepancies in the results. Some studies have found a connection between male infertility and mtDNA mutations in certain genes [29, 30], while others did not [31].

A recent study revealed five non-synonymous SNPs in nicotinamide adenine dinucleotide hydrogen (NADH) dehydrogenase 1 gene (ND1) located at nucleotides T3398C, T3821C, G4048A, T4169TT, and T4216C. Since secondary structure prediction of T3398C and T3821C proteins revealed a negative change in protein function, these alterations could result in sperm motility decline and male infertility [32].

Besides, two studies were conducted in our laboratory, the first study demonstrated that the frequencies of total mitochondrial variants in four mitochondrial genes (nicotinamide adenine dinucleotide hydrogen (NADH) dehydrogenase 1 (ND1), NADH dehydrogenase 2 (ND2), NADH dehydrogenase 5 (ND5), and NADH dehydrogenase 6 (ND6)) were negatively correlated with the percentages of sperm motility and ICSI outcomes, namely 13708 G>A, 4216T>C and 12506T>A [5, 33].

Mughal et al. discovered that mtDNA mutations had a major impact on sperm production, resulting in infertility, by influencing various sperm motility parameters [34]. Other studies reported the presence of a genetic mutation due to shifting in the SNP T4216C in fertile and infertile men [35, 36]. On the other hand, Zhang et al. pointed out that SNP C3398T is a lower risk of asthenozoospermia due to the low frequency and small sample size [37].

Earlier studies demonstrated that patients with the A3243G mtDNA mutation and large-scale mtDNA deletions were related to asthenozoospermia [38, 39]. Furthermore, subfertile men may have a variety of mtDNA mutations acquired during their lives [40], and these mutations may reach high levels within individual sperm progenitor cells, resulting in defective sperm motility and infertility [3].

## Conclusion

The current study showed a significant correlation between certain *MT-CYB* gene SNPs (rs527236194 (*P* = 0.0005), rs28357373 (*P* = 0.0439), and rs41504845 (*P* = 0.0038)) and the incidence of men’s infertility. However, the genetic alterations in these sites are synonymous which suggested the need for further studies to elucidate the molecular role of these SNPs on the gene expression level. In addition, more studies on the *MT-CYB* gene are required on different populations to reveal the exact role of the reported SNPs on men’s infertility.
